# Constrains in the articulation between profession, family, and personal life: a case study of the Portuguese police (PSP)

**DOI:** 10.3389/fsoc.2024.1341578

**Published:** 2024-06-04

**Authors:** Ana Rocha, Paula Espírito Santo, Ana Paula Ferreira

**Affiliations:** ^1^Institute of Social and Political Sciences, University of Lisbon, Lisbon, Portugal; ^2^Centro de Administração e Políticas Públicas and Institute of Social and Political Sciences, University of Lisbon, Lisbon, Portugal

**Keywords:** work, family, personal life, articulation, police officers (PSP)

## Abstract

The dangers and stresses of a police officer’s career cause physical and psychological distress which in turn cause considerable challenges for the integration of professional, family, and personal life. This study focuses on the conditions that influence this articulation between the personal, family, and professional lives of Portuguese Police (PSP^1^) officers. A questionnaire survey was applied to 414 police officers from 11 divisions of COMETLIS.^2^ At the empirical level, several models were tested to assess the articulation between the professional, family, and personal lives of police officers. The results highlight the influence of having young children, elderly people or other dependents living in the family home, as well as the employee’s age, on the job’s interaction with professional life. At a professional level, the variables shift work, professional category, years of service and working away from home were identified by the officers as conditioning factors in the work-family-personal life balance. Sex differences in the articulation of professional, family, and personal life were also observed.

## Introduction

In recent decades, western society has undergone several changes to the labour market and the family (Amorim, 2013) such as the increase in female education, the mass entry of women onto the labour market, and the fact that it is now more demanding and competitive (Oliveira, 2015). For couples with two household members in employment (Matias and Fontaine, 2012), along with the presence of new forms of family structure (Santos, 2011) and the participation of all family members in household activities and responsibilities, this has led to a redefinition of the roles traditionally attributed to men and women (Lima and Neves, 2011). As a result, individuals must seek out some form of balance to successfully navigate the competing demands of professional, family, and personal life (Santos, 2011; Perista et al., 2016). Currently, organisations have a legal^3^ obligation to provide working conditions that promote the link between professional, family, and personal life, for example, through family support policies being beneficial for workers, employers and society in general (GRAAL, 2000; Allen, 2001; Guerreiro et al., 2006; Teixeira and Nascimento, 2011). Moreover, organisations must implement informal practices that promote the family, such as, for example, support from supervisors (Thomas and Ganster, 1995).

The police profession is commonly considered to be both risky and stressful. The fact that employees frequently work in shifts and at unconventional times often leads to a disruption of rest and mealtimes and can also lead to everyday disagreements between family members and friends, as well as on special occasions. These circumstances can subject members of the police to great physical and psychological stress, which in turn result in considerable challenges to the task of balancing professional activity with finding time for personal and family life (Duxbury et al., 2021).

This article aims to analyse the balance between the professional, family, and personal lives of the Public Security Police (PSP)^4^ officers of the Lisbon Metropolitan Police Command (COMETLIS).^5^ Research on this professional group has increased, addressing topics such as professional stress and burnout (Branco, 2010; Luís, 2011; Oliveira and Queirós, 2012), and it is equally relevant to analyse the articulation between professional, family and personal life, especially at COMETLIS, a Command in which many officers begin their professional career and in which several of its members work away from home, making this articulation more difficult. Furthermore, from a social and academic point of view, understanding how these officers balance these three areas of their lives is also relevant. The articulation between these different spheres has been gaining prominence in the public debate. Leisure activities are now recognised as fundamental for the well-being of individuals, on a par with paid work and the family. These factors can be considered as key aspects in the lives of individuals (Maciel et al., 2008; Silva et al., 2010).

The initial question that guided the study was: which constraints do PSP officers from COMETLIS have in the articulation between professional, family, and personal life?

The general objective of this contribution is to learn about the family and professional conditions that influence the relationship between the officers’ professional, family, and personal lives. The specific objectives are:

- To identify the family and personal conditions that influence the relationship with the professional life of these officers;
- To assess the professional limitations in the articulation with the family and personal life of these officers.

To respond to these objectives, a questionnaire survey was carried out in 11 COMETLIS Divisions and included a sampling of 414 PSP agents, considering a universe of 2,633. The questionnaires were self-administered and provided by the institution via e-mail.

This article is split into three sections. The first, which follows the introduction, concerns the theoretical framework in which the themes are addressed, namely: the articulation between professional, family, and personal life, and secondly, the role of organisations in this articulation. In the second section, the methodological options that were used in this work are presented. In the third section, before the conclusion, a reading and discussion of the results is provided.

## Theoretical framework

### Articulation between professional, family, and personal life

There are several theoretical perspectives that explain the connection between the professional and family spheres. An example of this is the conflict perspective (Santos, 2011). Based on the work of Kahn et al. (1964), Greenhaus and Beutell (1985) defined the conflict between work and family as a form where the pressures of roles in the family and professional domains are incompatible in some respects. Conflict can have two dimensions: work–family conflict and family–work conflict. The work–family conflict occurs when work interferes with family activities. The family–work conflict is the opposite.

According to Greenhaus and Beutell (1985), there are three forms of work–family conflict: time-based conflict, tension-based conflict, and behaviour-based conflict. The first states that the time devoted to one role cannot be used in another role in life, i.e., the various roles played by an individual compete in terms of time. Regarding the police, for example, irregular working hours make it difficult to interact with the family and fulfil personal obligations (Youngcourt and Huffman, 2005). The tension-based conflict states that the tension created in one role makes it difficult to meet the demands of the other role, for example deaths on the job, of colleagues or even civilians. The last form of conflict, based on behaviour, argues that the role-specific patterns of behaviour may be incompatible with the expectations of another role. For example, the serious, professional demeanour required of a police officer whilst on duty, or the requirement to be fully alert to the dangers and circumstances of surroundings are not so necessary when with family or friends.

The mass entry of women onto the labour market and their ability to contribute financially to the household has meant that most couples now have two incomes, where historically there may have been only one. This of course means that the labour market and/or employers have had to adapt to this new reality. Furthermore, as the job market becomes more competitive and demanding (Oliveira, 2015), work itself, along with the search for better job opportunities in the future, sometimes requires workers to travel between cities (Silva, 2016) or even countries. Thus, family members may become geographically separated, a situation that occurs frequently in the PSP. After completing their training, police officers are placed in Commands and typically must work away from their family and friends. As a result, their commitment to the professional sphere can1 create difficulties in their involvement with their family (Teixeira and Nascimento, 2011).

In this work, the concept of family life includes family and domestic responsibilities. In the Portuguese context, these responsibilities are currently more equally shared amongst family members than in recent decades. The view of women being dedicated to the home and family has gradually been replaced by more equal perspectives between men and women (Torres, 2004; Núncio, 2008), however, women continue to devote more time to these responsibilities overall (Europeia, 2008; CIG, 2015; Ryle, 2018; Duxbury et al., 2021). Men and women find it difficult to balance professional, family, and personal life when they have small children (Perista et al., 2016). The provision of care to dependent adults is mostly provided by women, regardless of the age group or monthly income^6^ (Pimentel, 2011; Perista et al., 2016). These inequalities are associated with the stereotypes conferred on each sex (Borges, 2008). Maintaining these stereotypes can generate role conflicts and create asymmetries in the responsibilities of the two members of the couple in resolving these conflicts (Andrade, 2013).

Several contributions mention the balance between the professional and family sphere, leaving aside the personal sphere or encompassing it in the family domain. In this work, the personal sphere is associated with what individuals do with their free time, namely the activities of the officers when they are not at work. Free time is an essential element for quality of life, influencing personal and professional life (Cantorani et al., 2009). In addition to their professional and family responsibilities, individuals must have free time, as this provides physical and mental benefits (Shattell, 2010). Cantorani et al. (2009) add that free time activities are essential to break the routine, but also as an indicator of increased productivity at work. However, as the job market becomes increasingly demanding, it forces individuals to spend more time on professional activities to the detriment of those that give them pleasure (Beuter et al., 2005; Cantorani et al., 2009).

It becomes harder for women to balance their professional, family, and personal lives, as they end up effectively having a double working day, accumulating paid work during the day, and then carrying out domestic and care tasks once they return home (Núncio, 2008). As a result, women suffer more constraints regarding paid work than men (Perista et al., 1999), sometimes having to invest less in paid work to be able to fulfil their family commitments (Torres, 2000). In Portuguese society, over recent decades, the participation of women in the labour market has been increasing, although this change has not been equally reflected in the participation of men in domestic and care tasks (Perista, 2002; Torres, 2004).

### The role of organisations in the articulation between professional, family, and personal life

According to Marcello Caetano, the police is ‘(…) the administrative authority’s mode of action that consists in intervening in the exercise of individual activities that may endanger general interests, with the purpose of avoiding the production, expansion or generalization of the social damage that the laws seek to prevent’ (Caetano, 1990, p. 1,150).

The functions of the police are vast and comprehensive (Oliveira and Queirós, 2012). On the one hand it is a civil service profession associated with financial stability and of a humanistic nature (Poiares, 2005; Durão, 2021). On the other hand, it is associated with several risks: assault, death on duty, low pay, workplace accidents, night work, professional devaluation, inadequate and/or insufficient material means (Poiares, 2005), away from their origins, difficulties in career progression (Rodrigues, 2018), stress, danger, bureaucracy and shift work (Castelhano et al., 2010; Oliveira and Queirós, 2012), all of which can lead to physical illness, aggressiveness, depression, and suicide (Figueiredo-Ferraz et al., 2014), and contribute to work–family conflicts.

There are organisations that provide measures that help workers manage professional and non-professional responsibilities (Allen, 2001). These measures are often referred to as family-friendly policies (Albrecht, 2003) or family-friendly benefits. According to Marshall and Barnett, for an organisation to be characterised as ‘family friendly’, it must not simply implement family support policies, it must also implement informal practices and an organisational culture that promotes the family (Pimenta, 2011). It is important to note that the term ‘family friendly’ is not consensual (Lewis, 2001) as the term ‘friendly’ can be interpreted as a favour instead of a right. Therefore, in this work, the term ‘family support policies’ is preferred.

According to Allen (2001), the family benefits that organisations may provide can be split into two groups: flexible work agreements (flexible hours; compressed working week; telework and part-time work) and care structures for dependents (day-care centres in the workplace; subsidies for children; care consultations for children; paid maternity and paternity leave,^7^ as well as support for the elderly). Thomas and Ganster (1995) also add support provided by supervisors to the measures previously mentioned.

Since the PSP is an organisation with unique characteristics, not all the measures mentioned above are applicable. However, there are others that current employees or retirees and their families can benefit from, principally, the Social Services of the Public Security Police (SSPSP). The SSPSPs were created to fill a gap in the personal assistance of police officer. In the first years of their existence, these services were mainly devoted to sickness assistance, canteens, and social housing. Over the years, their fields of activity increased to include, amongst others, a social action office (GAS); a savings bank; subsidies; protocols and partnerships in various areas; and support facilities for retirees, officers, and their children (SSPSP, 2019).

In addition to the formal policies provided by the institutions mentioned above, informal policies are also important. Moreover, Thompson et al. (1999) and Allen (2001) mention that employees who perceived less family support from the organisation were more likely to suffer work–family conflicts, had a greater intention of leaving the organisation, were less satisfied at work and felt less organisational commitment, compared to employees who perceived their organisation as being more family-friendly.

### Methodological options

This work is a case study focused on the analysis of the career of COMETLIS PSP officers, for which quantitative and qualitative analysis was carried out. The data collection instrument was the questionnaire survey. We opted for the self-administered questionnaire which was sent to the officers’ service e-mail address provided by the institution, after approval by the Commander of COMETLIS. Participation in the questionnaire was voluntary and participants were guaranteed that the answers were anonymous and confidential, only to be used in the context of this study. At the empirical level, the Allen (2001), Carlson et al. (2000) and Neto (2014) models were tested to assess the articulation between the professional, family, and personal lives of police officers.

The questionnaire is composed of 51 questions (Annex 1), 37 of which are split into 3 groups. The first group of questions, G1, concerns work–family conflict and has 18 questions (1–18) (Carlson et al., 2000). The second group, G2, refers to the family support work environment and has 14 questions (19–32) (Allen, 2001). The answer options for these two groups of questions were made using a Likert scale that ranged from strongly disagree (1) to strongly agree (6). The third group of questions, G3, corresponding to the influence of shift work on family and personal life, contains five questions (33–37), adapted from Neto (2014), with the following answer options: Never; Almost never; Sometimes; With some frequency; Very frequently. The original instruments were translated and adapted to Portuguese considering the realities of the institution being analysed, and a pre-test was carried out to check if they were appropriate. The remaining questions and variables are sex; age; marital status; workplace (division); professional category; years of service; location; dependent underage children; carer of elderly or dependent persons, and free time. Respondents were also asked to leave suggestions for their organisation on facilitating measures to balance their professional, family, and personal lives. The questionnaire was conducted between July and September 2018, and was sent to 2,633 PSP officers who were, at the time, working in 11 COMETLIS Divisions: 1st; 2nd; 3rd; 4th, and 5th (Lisbon); Amadora; Cascais; Loures; Oeiras; Sintra and Vila Franca de Xira. The questionnaire was sent to 2,633 agents and the response rate was 19.1%. Only 414 questionnaires were considered valid. The effective response rate was 15.7%.

For the characterisation of the sample, descriptive statistical measures were used (frequencies and cross tabulation).

For the G1 and G2 groups, an exploratory factor analysis using Principal Component Analysis (PCA) was carried out, and inter-factor correlations were made. To examine the relationships between characterisation variables and factors, non-parametric tests were conducted (Mann–Whitney, Kruskal-Wallis) as normality was not observed for any item. Content analysis was carried out for suggestions left by the respondents concerning the balance between professional, family, and personal life. Four categories were considered: (i) working hours; (ii) work conditions and value placed on the workforce; (iii) transfers between district commands; (iv) family support policies and initiatives.

## Results

Analysis revealed that 89.9% of the participants were men and 10.1% were women; 48.4% were between 31 and 40 years old and 10% between 51 and 60 years old; 73.4% were married or in a partnership; 19.6% were single and 7% were divorced or separated; 46.9% had underage children (up to 12 years old), and of these, 94.3% reported that they had custody of their children; 14.7% had elderly or other dependents.

When asked about how they spent their free time, respondents had 10 response options of which they could select up to three. According to the data, the leisure activity most frequently indicated by the officers was ‘Sports activities’ (69.6%) and the least indicated was ‘Reading’ (9.9%). In the ‘Other’ grouped categories, which only 50 respondents selected, ‘Time for yourself, family and friends’ was the most frequent with 30% of the responses, followed by ‘Domestic and care activities’ with 26%.

59.9% of respondents were officers and 40.1% were principal officers with Loures Division being the most participative with a response rate of 18.8%. Regarding years of service, 26.5% of the employees had between 10 to 14 years of service and only 6.3% of participants had more than 30 years of service. 43.2% of respondents were working away from home, and of these, the majority (50.3%) were from the North of the country.

Descriptive statistics for the 32 work–family conflict items (G1) and family support work environment (G2) are given in Table 1 of Annex 2.

**Table 1:** Distribution of questionnaire variables, by associated factors and study groups.

Construct	Factors	Questionnaire items	Cronbach’s alpha
G1	F1	13; 14; 15; 16; 17; 18	0.897
G1	F2	1; 2; 3; 7; 8; 9	0.884
G2	F3	19; 20; 24; 25; 28; 29; 32	0.827
G1	F4	10; 11; 12	0.926
G1	F5	4; 5; 6	0.806
G2	F6	21; 22; 26; 27	0.680

Source: the authors.

### Analysis of the relationship between factors and family contexts

PCA analysis was carried out for G1 and G2, the KMO was of 0.848 and the Bartlett test of sphericity *p*-value was less than 0.001. The distribution of items amongst factors (Table 1) was as follows: G1 has four factors (F1; F2; F4; and F5) and all original items were considered; G2 group has two factors (F3 and F6) and items 23, 30 and 31 were not considered for this construct. Factor loadings for each item are given in Table 1 (see Annex 3 for more detail), and the option to keep two variables with loadings lower than 0.5 in the factors is related to their importance for the theoretical discussion. A reliability analysis was carried out for each factor and Cronbach’s alpha showed the factors with acceptable reliability as shown in Table 1. Each factor was assigned a designation according to the items it contained, to facilitate interpretation as follows:

F1—behaviour at home vs. behaviour at work;
F2—time and tension at work interfere with the family;
F3—work priority vs. family priority;
F4—family tension interferes with work;
F5—family time interferes with work;
F6—separation between professional, family, and personal life.

The item distribution is quite like that observed by Carlson et al. (2000), and factors F4 and F5 contain the same items. Contrary to the results from Allen (2001) the items in G2 group were split into two factors.

Factor 3 ‘work priority vs. family priority’ is related to factor 6 ‘separation between professional, family, and personal life’ (r = 0.489, *p*-value < 0.001) and factor 4 ‘family tension interferes with work’ is somehow related to factor 5 ‘family time interferes with work’ (r = 0.491, *p*-value < 0.001).

To analyse the existence of a relationship between the studied variables and the factors from the two constructs, non-parametric tests were conducted. The results show that:

- For the ‘children in their custody’, ‘marital status’ and ‘division where they perform their duties’ conditions, no relationship between the different groups and factors was found;
- Considering the ‘time and tension at work interfere with the family’ factor, a connection with the ‘having underage children’ condition was identified, with a p-value of 0.026 and a considerable difference in the mean rank;
- For those who have ‘elderly or other dependents’, the analysis shows a relationship with the ‘separation of professional, family and personal life’ condition. A significant difference in the mean rank was obtained, and the p-value (0.002) also shows the existence of a relationship;
- There is a significant difference between ‘men’ and ‘women’ concerning the ‘family time interferes with work’ condition (*p*-value = 0.039). For men, the mean rank is higher;
- The ‘professional category’ is related to three factors: ‘family tension interferes with work’ (*p*-value = 0.008); ‘family time interferes with work’ (p-value = 0.014) and ‘separation of professional, family and personal life’ (*p*-value = 0.038). The higher the position, the higher the mean rank, which means more interference;
- The ‘working away from home’ condition is related to ‘time and tension at work interfere with the family’ (*p*-value = 0.000), as already expected;
- Analysis reveals that ‘years of service’ has a relationship with three factors: ‘family tension interferes with work’ (*p*-value = 0.002); ‘family time interferes with work’ (*p*-value = 0.029) and ‘separation of professional, family and personal life’ (*p*-value = 0.020);
- The ‘age’ of the respondents is also related to the ‘family tension interferes with work’ (*p*-value = 0.003) and ‘separation of professional, family and personal life’ (*p*-value = 0.019) conditions.

### Analysis of the influence of shift work on family and personal life

The analysis reveals that officers consider that shift work ‘Very frequently’ interferes with their family life (50.5%) and personal life (44.4%). On the other hand, Officers consider that ‘Sometimes’ family and friends accept working hours (45.7%), whilst 38.4% said that ‘Sometimes’ shifts allow them to have the time they consider necessary for family life and personal life (41.5%).

No significant differences between ‘men’ and ‘women’ were found for any of the items under analysis in the G3 group.

The questionnaire also contained an open-ended question allowing officers to suggest measures for their organisation to help facilitate the articulation between professional, family, and personal life. 35% of respondents left suggestions on this issue. Given the nature of the variable, six categories were created to accommodate the suggestions. The analysis highlights:

- Working hours: the need for reduced and flexible working hours, as well as the need for more time off to allow better articulation between professional, family, and personal life;
- Working conditions and value placed on the workforce: it would be important to improve working conditions not only of facilities, but also of work materials and, in that scope, to increase the number of employees. There is a significant work overload which often leads to employees being seen simply as numbers by their hierarchical superiors;
- Transfers: it would be essential to reduce transfer waiting times between Commands. Working in a Command close to family and friends contributes to increased well-being and allows better articulation between the different spheres;
- Family support policies and initiatives: the creation of day-care centres for their children, and for working hours to be complemented by physical activity and initiatives to improve corporate spirit.

## Discussion

The police profession is associated with financial stability, a reassuring future, a humanistic nature (Poiares, 2005; Durão, 2021), but is also commonly considered to be a risky and stressful one, often associated with high levels of physical and psychological stress, making it difficult to articulate professional, family, and personal life. It is therefore important to understand how officers balance the different spheres of their lives. The results of this study demonstrate officers’ opinions on the existence of work-family and family–work conflict.

The sample in this study consists mainly of men (89.9%) in the 31–40 age group (48.4%) and with 10–14 years of service.

In the ‘With regard to your personal life, how do you spend your free time?’ variable (tick up to three options)’, the answer with the highest values was Sports activities (69.6%), which makes sense, as this profession generates high levels of physical and psychological stress, and physical exercise helps prepare the body for everyday situations and frees the mind of the stress inherent to the profession. In the ‘Other. Which one?’ option, the most frequent response category is ‘Time for yourself, family, and friends’ (30%), demonstrating the importance and fundamental nature of time spent with their nearest and dearest. This variable included leisure activities, however, some of the free time these individuals have is spent on household and care tasks (26%) and not on activities that fulfil and satisfy them, and as Cantorani et al. (2009) mention, activities carried out in leisure time are essential for quality of life, influencing personal and professional life and providing physical and mental benefits too (Shattell, 2010).

The perception of the interference of time and tension experienced at work in the family is different between officers with young children (up to the age of 12) and those who have either no children or children of legal age. Since they are dependent, minors need more care and attention. The time spent at work and the tension associated with it can generate sources of conflict which interfere with the management of family and personal life. For the police, irregular working hours make it difficult to interact with family and fulfil personal obligations (Youngcourt and Huffman, 2005).

Officers with elderly or other dependents find it more difficult to balance professional, family, and personal life. They often feel that family and personal matters must stay at home, so they make a greater effort to separate professional, family, and personal life. This is an increasingly relevant topic, as the population is ageing, and more families are facing the responsibility of having to take care of their elderly relatives. Generally, organisations are not prepared for this factor yet, and the officers who must provide this type of care may experience difficulties in managing their professional and family spheres. Organisations still see these spheres as separate, however, they strongly influence each other and cannot be separated (Santos, 2011).

Policemen and policewomen had different opinions on the interference of family time at work. Our results show that men feel that family time interferes more with work than women. Considering that the PSP is a so-called ‘male organisation’, policewomen often feel they are under added pressure to react to this and to prove that they can articulate the various spheres of life without difficulty. Contrary to what is reported in the literature, it is more difficult for women to articulate their professional, family, and personal lives, sometimes having to invest less in the work sphere to be able to accompany the family (Torres, 2000). Men are ‘allowed’ to let professional life interfere in family life, whilst women are expected to put family life first. This is the result of the cultural heritage passed down through the generations (Loureiro and Cardoso, 2008). Although there has been an increasing participation of women in the labour market in Portugal in recent decades, this change is not equally reflected in the participation of men in domestic and care tasks (Perista, 2002; Torres, 2004). In general, women have a double working day (paid work and domestic and care tasks) and thus family overload frequently restricts their ability to carry out their professional duties as expected.

The different professional categories (officer and principal officer) had different views on the separation of professional, family, and personal life, and on the interference of family time and tension at work. When compared with lower ranking officers, senior officers, who in most cases are older with already formed families, consider it more important to separate the professional, family, and personal spheres as they are more likely to consider that time and tension in the family interfere with work. The tension and the time spent managing family responsibilities can create difficulties in articulating professional life. These difficulties can influence the performance of professional duties and interfere with career progress.

Officers working away from home have a different opinion on the interference of time and work tension in the family from those who work close to home. Officers who work away from home obviously spend less time in their area of residence or its immediate vicinity. This is why they feel a greater conflict between professional and family life, as they are not present in the family’s day-to-day activities, but only on breaks or during holidays, which compromises relationships with their friends and relatives. Even though we are living in a digital age where physical distances can be bridged using video calling for example, face-to-face contact remains healthier and more important. Transfers between Commands is a further difficulty to be solved, as the waiting time between transfers is very long.

Younger officers have a different opinion from older officers on the interference of family tension at work, as well as on the separation of professional, family, and personal life. As they usually have a family, older officers consider that family tension interferes more with work, and because they consider that family matters should be separated from their professional life, they are better at keeping those spheres separate. As many of the younger officers have not yet started a family, the tension between the two spheres is less of an issue. All officers consider that there must be a separation of professional, family, and personal life, but as the years of service increase, officers consider this separation increasingly important because older officers believe that family time and tension can interfere with work.

There were no significant differences between sex relative to shift work making it difficult for officers to balance their professional, family, and personal life. Shift work is a constraint on the balance between professional, family, and personal life, as it generates fatigue and impairs the performance of family responsibilities and contact with family and friends (Castelhano et al., 2010; Perista et al., 2016). The literature also states that women are more likely to consider quitting operational activities in favour of more administrative functions (Durão, 2021). Here, the physical and psychological risks are reduced, and the schedules are relatively fixed, meaning it is easier to balance the various spheres of life (Castelhano et al., 2010; Caroly, 2011). When men choose these functions, their superiors consider them to be weak (Caroly, 2011). According to the study by Caroly (2011), some women choose to work night shifts, so that they can carry out family tasks during the day, but this leads to sleep disorders and fatigue. However, in our study we were unable to verify that female officers who work in shifts find it more difficult to balance the different spheres than male officers. Through the results of the questions in the ‘influence of shift work on family and personal life’ group (G3) and the question to which they could provide suggestions for measures that help facilitate articulation, it is also visible that officers feel shift work interferes with the articulation between professional, family, and personal life.

As Greenhaus and Beutell (1985) state, the conflict between work and family can have two meanings: work–family conflict and family–work conflict, and three forms: time-based, tension-based, and behaviour-based conflict. According to our results, the conflict arose in both directions (between work and family and family and work), and men, officers who have minors, those working away from home, the number of years of service and their professional category, all have an impact on the ability to find balance. Moreover, the officers in those circumstances feel that time dedicated to one life role cannot be spent in another, and that the different professional, family, and personal spheres compete in terms of time, as described in the time-based conflict. Furthermore, age, having underage children, being out of service, the number of years of service and the professional category can all create tension, which makes it difficult to meet the requirements of other professional, family, and personal responsibilities, as mentioned in the tension-based conflict.

Some of the family support policies suggested by the officers are in line with the literature. Thomas and Ganster (1995), Allen (2001), and Albrecht (2003) suggest measures such as: flexible working hours, family assistance measures, parental leave, and support from supervisors. Russell et al. (2009) consider that the reduction and flexibility of working hours reduces pressure at work and work–family conflict. It is essential for the PSP to take these needs into account, as these measures can help officers articulate professional, family, and personal responsibilities.

## Conclusion

Based on the results obtained, and in response to our initial question, COMETLIS PSP officers articulate professional, family, and personal life with constraints at a family level and limitations at a professional level. Men consider that time with family interferes more with work than women. This balance could be improved if there were facilitating measures by the organisation, as the officers stated in the work.

Having minors, elderly or other dependents, as well as age, were the family conditions that most officers consider interfere most with the articulation with their professional lives. Following on from this study, it would be beneficial for the PSP to create day-care centres close to the Divisions, with schedules adapted to its workforce. Regarding elderly dependents, the PSP could create more infrastructure, for example a care home or residential unit, to offer support and extend these services to the family.

Shift work, professional category, years of service and being on duty, were the professional limitations identified by officers as being those that impact the articulation with their family and personal lives. Greater speed in completing transfers between Commands and an adaptation of working hours to shorter shifts and working weeks are factors the PSP should consider.

We conclude that male PSP officers consider that family time interferes more with their work than female officers. Female officers defend themselves in these types of situations, wanting to demonstrate that they are capable of easily articulating the various spheres of their lives. This is not in line with the literature however, because despite the increasing participation of women in the labour market, the same is not true of the participation of men in domestic and care tasks. To be able to attend to their families, apparently women tend to invest less in their work, therefore making it more difficult for them to articulate professional, family, and personal life, whereas for men, professional life can interfere in family life. This can be seen frequently because of the cultural heritage passed down over time from generation to generation (Loureiro and Cardoso, 2008).

Regarding methodological limitations, this study was applied to COMETLIS officers only, and therefore cannot be extrapolated to the wider PSP. Thus, in future research, it would be useful to replicate the questionnaire to all Divisions and professional categories.

## Data availability statement

The raw data supporting the conclusions of this article will be made available by the authors, without undue reservation.

## Author contributions

AR: Conceptualization, Data curation, Formal analysis, Investigation, Methodology, Writing – original draft, Writing – review & editing. PE: Conceptualization, Data curation, Formal analysis, Investigation, Methodology, Writing – original draft, Writing – review & editing, Supervision. AF: Data curation, Formal analysis, Investigation, Methodology, Supervision, Writing – original draft, Writing – review & editing.

## Footnotes

^3^As described in Article 127, paragraph 3 of Law no. 7/2009 (Diário da República, 2009).

^4^In the PSP, the police members are organised into three careers. The career analysed in this work is that of officer, which is the entry-level career. This career includes three subcategories, according to the professional evolution.

^5^PSP is organised by regional, metropolitan and district commands. The Commands are sets of Divisions of an area of the country, and Divisions, in turn, are made up of police stations. For this study, Lisbon was the selected area. The command, COMETLIS, has 16 Divisions, and 11 participated in this work.

^6^Draft Law no. 186/XIII/4th is under debate in the Parliament which “(…) Establishes measures to support informal caregivers and regulates the rights and duties of the caregiver and the person cared for (…)” (Assembleia da República, 2019).

^7^The term ‘maternity and paternity leave’ was used as described in the original article (Allen, 2001), although in Portuguese legislation the term parental leave is the adopted term.
