# Supplementary material for: Constrains in the articulation between profession, family, and personal life: a case study of the Portuguese police (PSP)
Source: Front Sociol. 2024 Jun 4;9:1341578. doi: 10.3389/fsoc.2024.1341578 (PMC11184944; doi:10.3389/fsoc.2024.1341578)

Annex 1 – Questionnaire


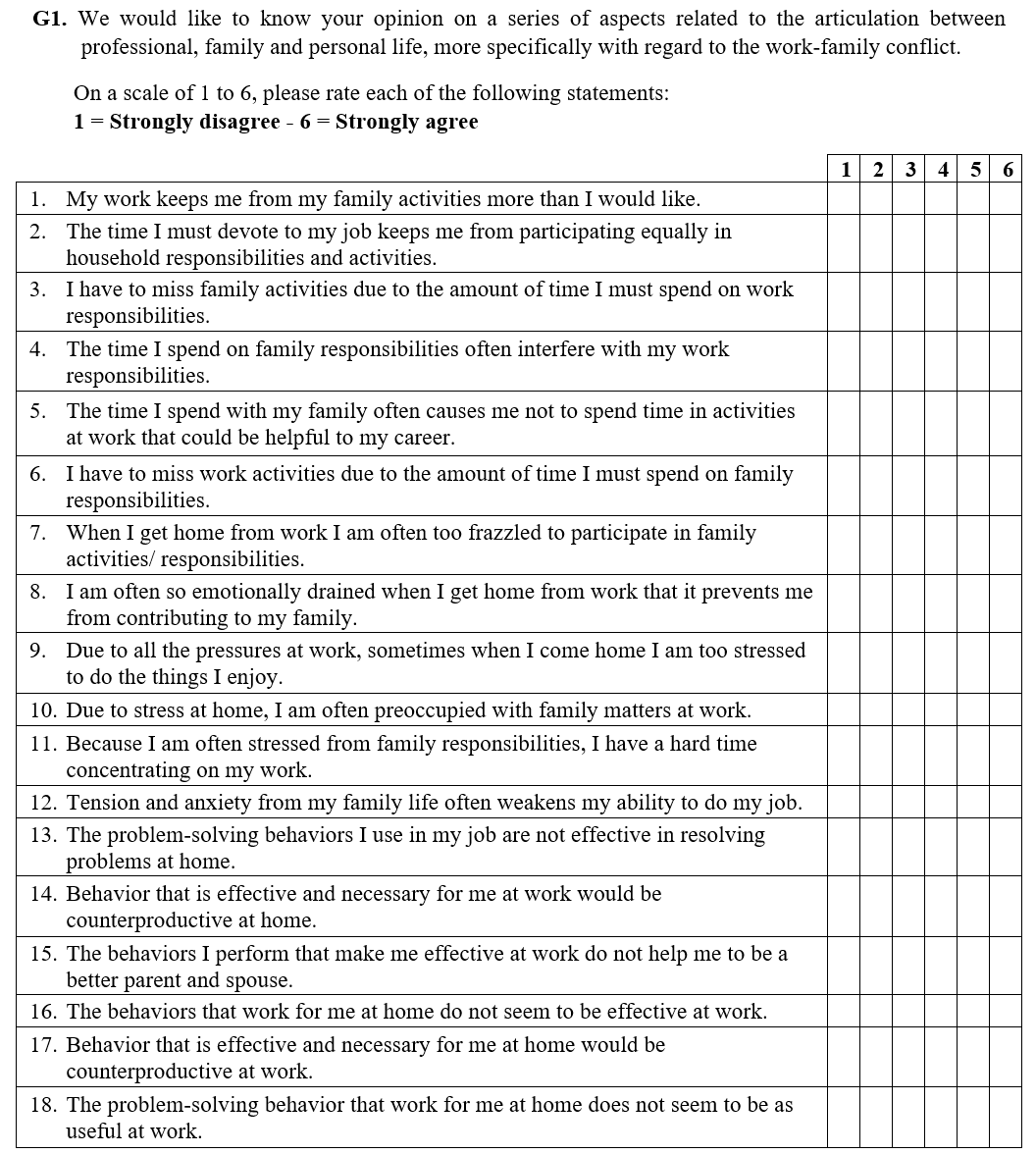


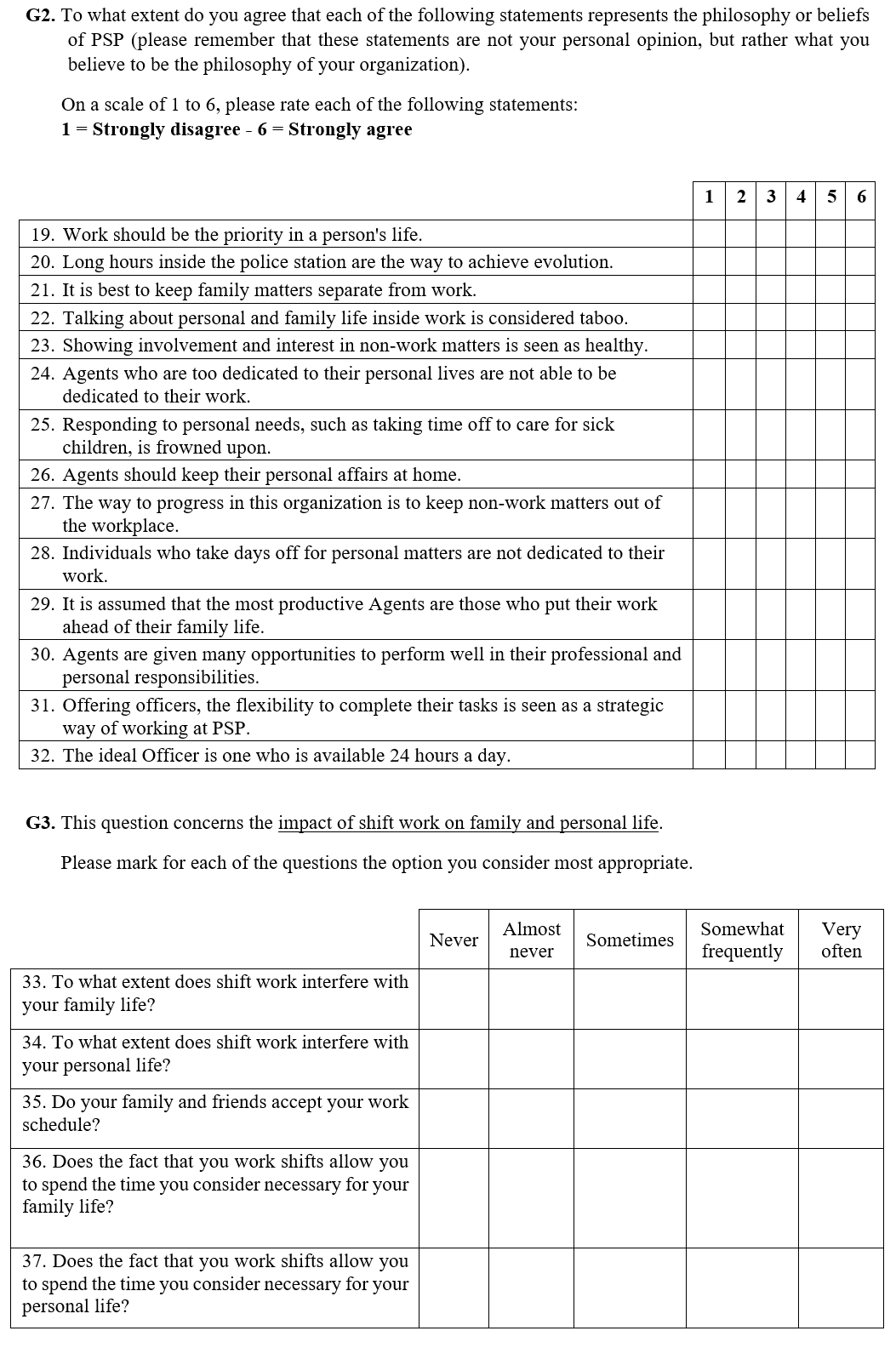


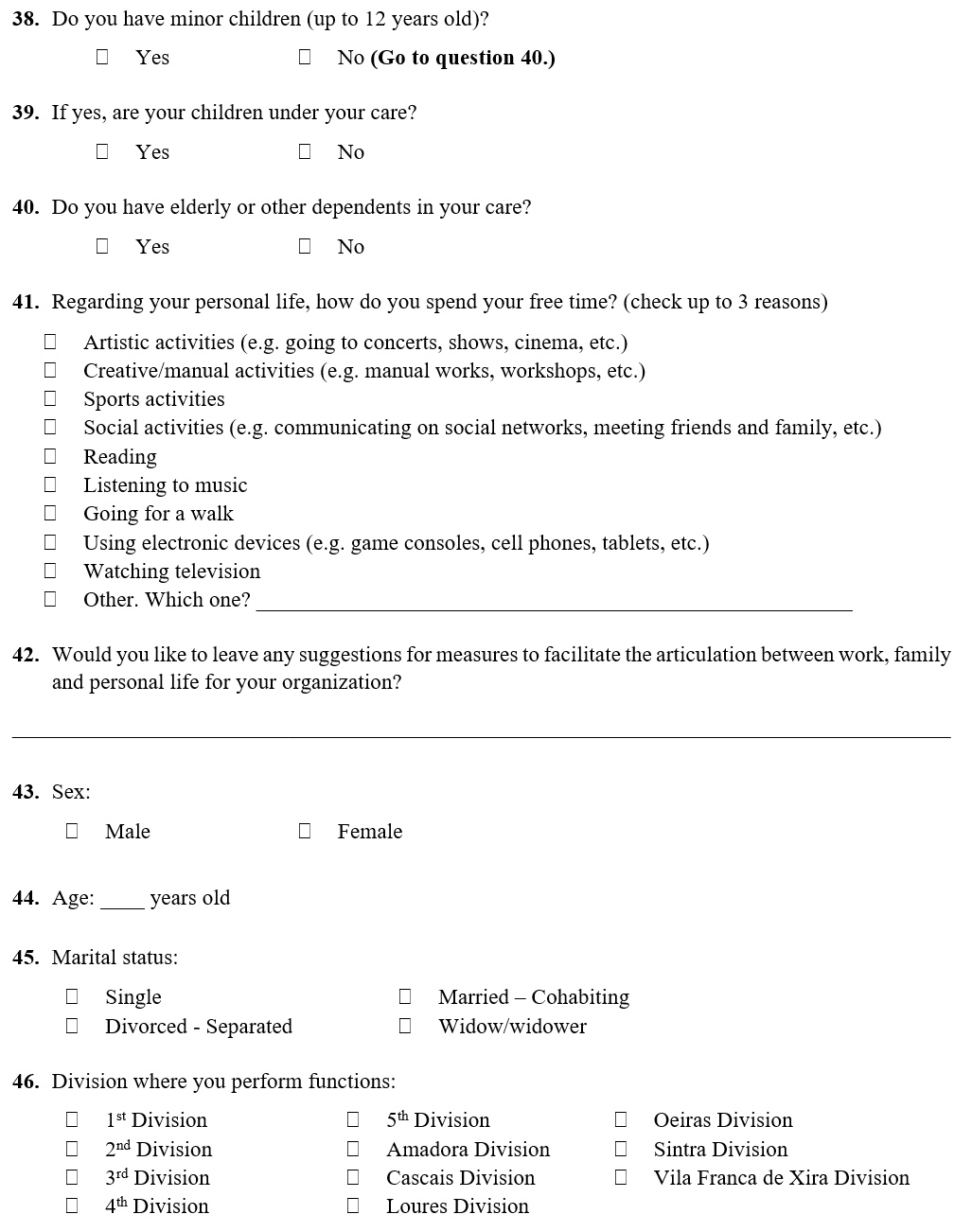


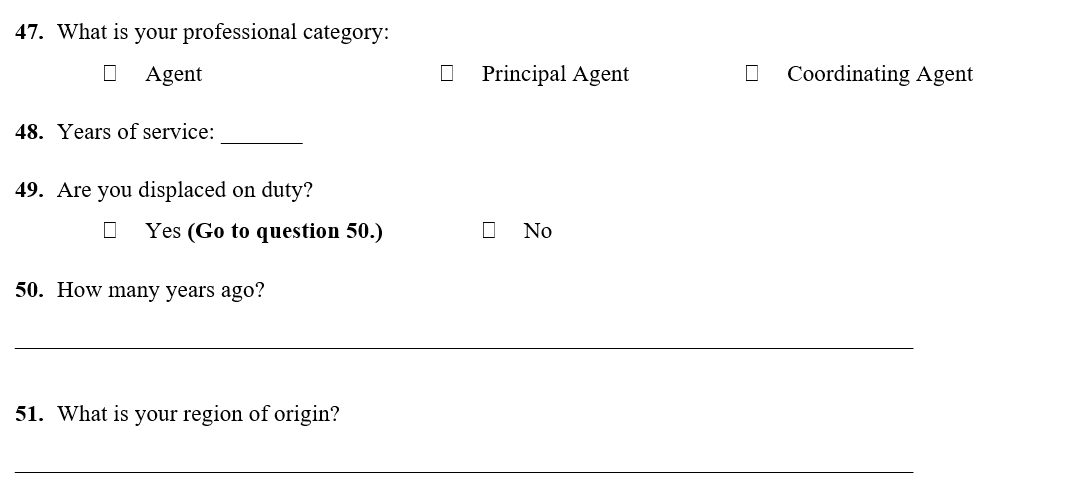


Annex 2 – Descriptive statistics

Table 1 – Mean, standard deviation and median of the 32 items in groups G1 and G2.

Source: the authors


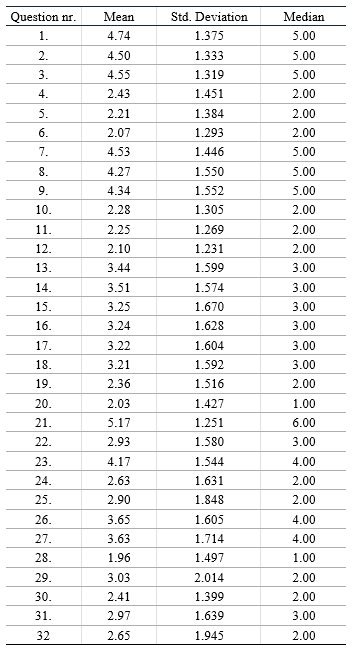


Annex 3 – PCA analysis loadings

Table 1 – Rotated component matrix, factor loading (items from G1 and G2).

Source: the authors


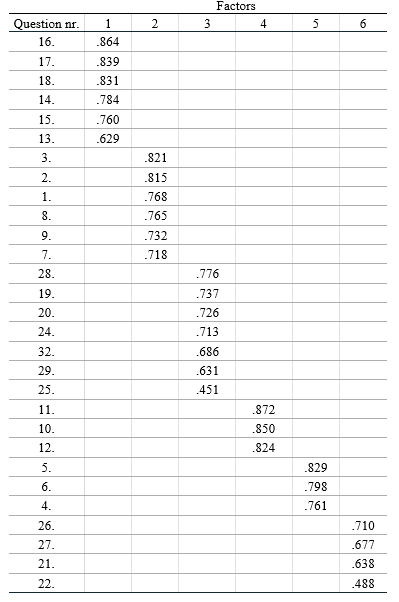

Supplement: Supplementary file 1 [file Data_Sheet_1.docx]
